# Supplementary material for: Spatial distribution of mammography adherence in a Swiss urban population and its association with socioeconomic status
Source: Cancer Med. 2018 Oct 25;7(12):6299–307. doi: 10.1002/cam4.1829 (PMC6308042; doi:10.1002/cam4.1829)
Supplement: Supplementary file 1 [file CAM4-7-6299-s001.pdf]

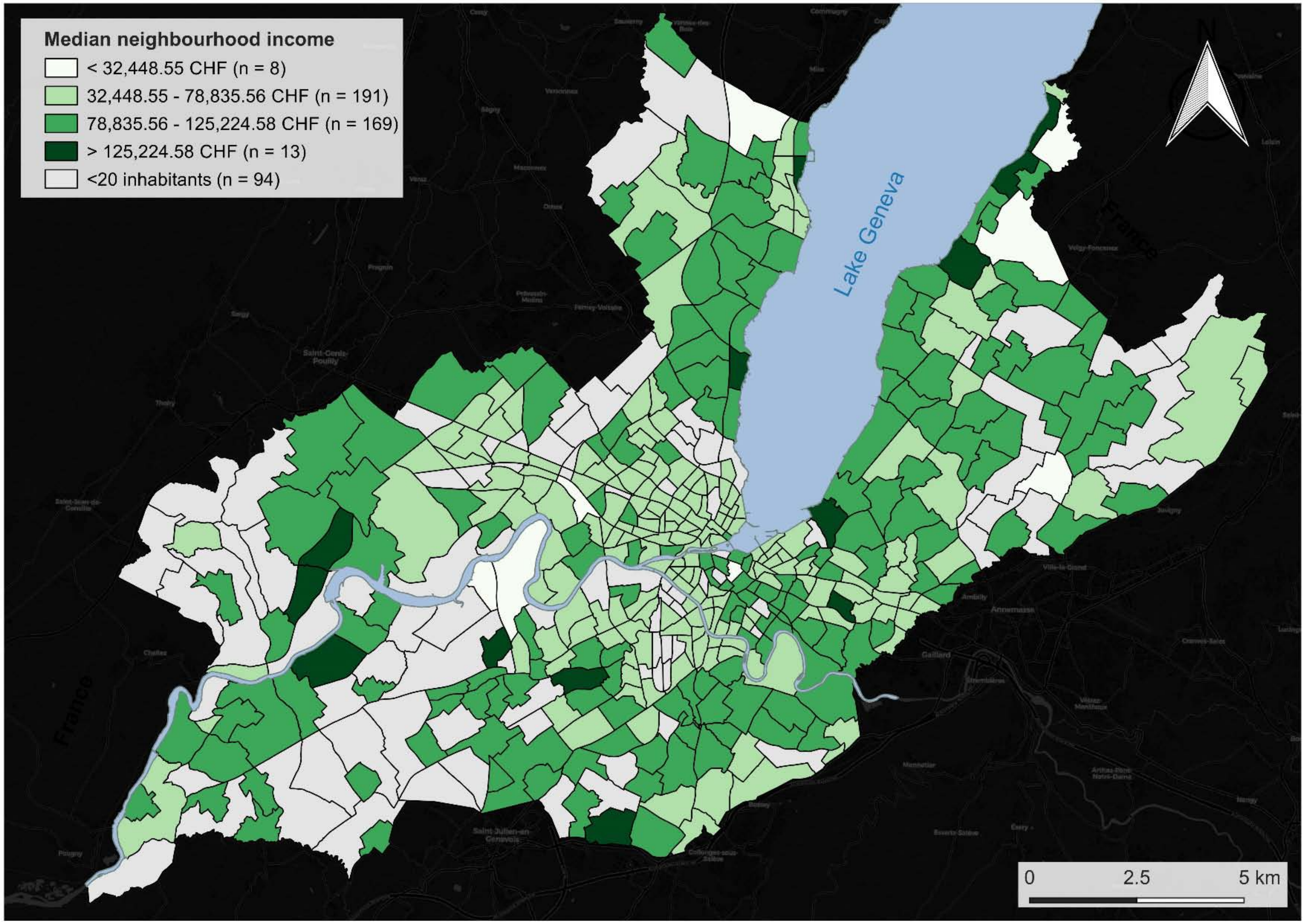

Supplementary figure 1 – Distribution of mean yearly income by neighbourhood in the state of Geneva, in CHF. Data were obtained from the 2013 Geneva census (www.ge.ch/statistique, Geneva Statistics Office).
